# Adsorption Mechanism of Short Hydrophobic Extended Anionic Surfactants at the Quartz–Solution Interface

**DOI:** 10.3390/ma18235392

**Published:** 2025-11-29

**Authors:** Linlin Zhang, Zhicheng Xu, Zhiqiang Jin, Wangjing Ma, Lei Zhang, Lu Zhang

**Affiliations:** 1 Technical Institute of Physics and Chemistry, Chinese Academy of Sciences, Beijing 100190, China; zhangll-1980@163.com (L.Z.); zhchxu@mail.ipc.ac.cn (Z.X.); jinzhiqiang@mail.ipc.ac.cn (Z.J.); wjma@mail.ipc.ac.cn (W.M.); zhanglei@mail.ipc.ac.cn (L.Z.); 2 University of Chinese Academy of Sciences, Beijing 100049, China

**Keywords:** short hydrophobic extended anionic surfactants, quartz, adsorption, PO group, EO group

## Abstract

This study elucidates the interfacial adsorption behavior of a series of short-chain extended anionic surfactants C_8_P_x_E_y_C (sodium ethylhexyl polyoxypropylene x-polyoxyethylene y-carboxylate) on quartz surfaces, with a focus on the regulatory role of polyoxyethylene (EO) and polyoxypropylene (PO) groups. The results indicate that there is a two-stage adsorption process for C_8_P_x_E_y_C molecules on the quartz surface. In the first adsorption stage, C_8_P_x_E_y_C molecules adsorb at the interface primarily via hydrogen bonding with their hydrophobic tails oriented to water and thereby reducing quartz hydrophilicity before the critical micelle concentration (CMC). The second adsorption stage appears at the CMC, and a saturated monolayer forms via hydrogen bonding. When further increasing the concentration, C_8_P_x_E_y_C molecules interact with the pre-adsorbed monolayer by hydrophobic interactions to establish a loose bilayer structure. As the hydrophilic heads of C_8_P_x_E_y_C orient to water, the surface hydrophilicity of quartz enhances after CMC. The PO/EO chain length critically governs the adsorption behavior of C_8_P_x_E_y_C. Specifically, longer PO chains enhance the molecular size and hydrophobicity of C_8_P_x_E_y_C, leading to a decreased saturation adsorption amount. Conversely, longer EO chains improve the hydrophilicity of C_8_P_x_E_y_C, promoting C_8_P_x_E_y_C molecules to stay in water and consequently decreasing the interfacial adsorption amount of C_8_P_x_E_y_C. This structure–activity relationship of C_8_P_x_E_y_C molecules guides the design of extended surfactants for applications requiring precise wettability control.

## 1. Introduction

The wettability of solid surfaces is a pivotal factor governing the performance of various industrial applications, such as detergency, dyeing, flotation, painting, and lubrication [1,2,3]. In oil exploitation, the wettability of rocks in the reservoir determines the oil recovery efficiency. The ability of surfactants to modify solid wettability has been extensively studied for decades and holds significant technological importance [4,5]. The wettability of solid surfaces depends on the adsorption amount of surfactants and their molecular orientation on the adsorption layer [6,7,8].

Quartz has a three-dimensional network structure formed by interconnected silicon-oxygen tetrahedra. Additionally, the quartz surface possesses numerous active silanol groups, endowing quartz with a high surface free energy of approximately 76.4 mN/m^2^. Quartz exhibits strong hydrophilicity in its native state. Therefore, the quartz surface is readily wetted by the aqueous phase through hydrogen bonding [9]. This characteristic gives it an advantage in applications that require strong wettability, such as coating substrates and composite material interface bonding. However, the inherent hydrophilicity of quartz also limits its use in applications requiring hydrophobicity or controlled wettability, such as antifouling and self-cleaning applications. Therefore, wettability modification has become a key strategy to expand the utility of quartz. This approach can transform quartz from a hydrophilic and easily wettable surface to a hydrophobic surface. It even allows quartz for the precise tuning of the contact angle through molecular design, meeting the customized demands for interfacial wettability in emerging fields like flexible devices and marine antifouling materials. The wetting behavior of quartz is not only influenced by surfactants’ adsorption at air–water, quartz–water, and air–quartz interfaces but also determined by the characteristic and activity of the adsorption layer.

Studies have shown that the adsorption mechanisms of different types of surfactants vary significantly on quartz surfaces. Cationic surfactants adsorb on quartz surfaces through electrostatic interactions to form hydrophobic monolayers or bilayer structures. Consequently, the quartz surface is hydrophobically modified, and the strong hydrophilicity of the quartz surface is weakened. Zhang et al. [10] found that the surfactants hexadecanol glycidyl ether ammonium chloride (C16PC) and guerbet alcohol hexadecyl glycidyl ether ammonium chloride (C16GPC) self-assemble into an oriented monomolecular layer on the quartz surface. In addition, these surfactants can further assemble into bilayer structures as the concentration increases.

Anionic and nonionic surfactants primarily adsorb on the surface of quartz by Lifshitz–van der Waals interactions. The group of Zdziennicka examined the effects of anionic, cationic, and nonionic surfactants on quartz surfaces’ wettability. It was observed that a significant linear correlation existed between adhesion tension and surface tension for nonionic and anionic surfactant solutions [11,12].

As for zwitterionic surfactants, they can adsorb on quartz surfaces through electrostatic attraction between positively charged sites in their headgroups and negatively charged sites on the quartz surface. Hu et al. [13] found that the contact angles of carboxybetaines (alkyl carboxylbetaine (ACB) and ditolyl substituted alkyl carboxylbetaine (BCB)) on quartz surfaces remained stable over a wide concentration range, and the contact angles only began to rise when the concentration of ACB and BCB solutions exceeded their critical micelle concentration (CMC) values. Li et al. [14] found a lower adsorption amount for zwitterionic surfactants (hexadecanol polyoxyethylene(3) glycidyl ether glycine Betaine (C_16_(EO)_3_PB) and guerbet alcohol hexadecyl polyoxyethylene(3) glycidyl ether glycine Betaine (C_16_G(EO)_3_PB)) on the quartz–water interface versus the air–water interface. This may be attributed to the parallel orientation of C_16_(EO)_3_PB and C_16_G(EO)_3_PB molecules at the quartz–water interface due to interactions between oxyethylene (EO) groups and the quartz surface, resulting in a steric hindrance effect. Experiments further confirmed that these surfactants can form transient saturated adsorption films on quartz surfaces. However, on account of the large size of their polar groups, these surfactants can hardly adsorb further at the quartz interface after CMC.

Extended surfactants exhibit unique interfacial properties derived from their distinctive molecular structure [15,16]. A key structural feature of the extended surfactant is the insertion of nonionic spacer groups, including EO and oxypropylene (PO) units, between its conventional hydrophobic tail and its hydrophilic group [17,18]. According to Chen et al. [19], the coiling tendency of PO chains at interfaces promotes a rugby ball-like molecular arrangement for extended surfactants. This unique structure endows extended surfactants with low interfacial tension, good salt tolerance, and thermal stability [20,21]. Therefore, extended surfactants are promising for applications in enhanced oil recovery, separation and extraction, and fabric cleaning. Do et al. [22,23] were the first to propose that low-concentration aqueous solutions of extended surfactants could be used to extract vegetable oils. It was found that extended surfactants effectively extracted most natural triglycerides after achieving ultralow interfacial tension. Based on the microemulsions of extended surfactants, Attaphong et al. [24] investigated low-temperature detergency of triglycerides and vegetable oils.

However, there are few reports about short hydrophobic extended anionic surfactants. By measuring the surface tension as well as contact angles, the adsorption mechanism of extended anionic surfactants with short hydrophobic chains (C_8_P_x_E_y_C) on the quartz surface is investigated in this work. Additionally, this investigation explores how the PO/EO chain length modulates the interfacial behavior of C_8_P_x_E_y_C surfactants. The results are expected to offer valuable insights for the molecular design of extended anionic surfactants aimed at precise wettability control of quartz surfaces.

## 2. Materials and Methods

### 2.1. Materials

**Scheme 1:**
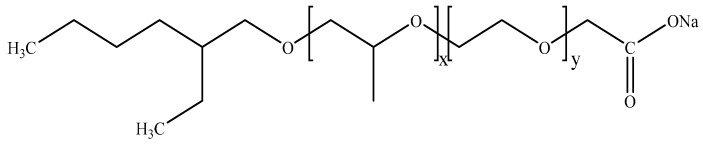
Structures and abbreviations of C_8_P_x_E_y_C molecules.

We synthesized the C_8_P_x_E_y_C extended surfactants (Scheme 1) in our laboratory. Their purity was confirmed to be ≥90% by high-performance liquid chromatography (HPLC). All inorganic reagents used in this work were of analytical grade. Ultrapure water (resistivity ≥18.2 MΩ·cm) served as the solvent for all solution preparations.

### 2.2. Apparatus and Method

#### 2.2.1. Surface Tension Measurement

The C_8_P_x_E_y_C solutions’ surface tension was tested under ambient pressure using a DCAT21 tensiometer (Dataphysics Instruments GmbH, Feldstadt, Germany) via the Wilhelmy plate method. To remove impurities, the platinum plate was first rinsed with pure water and then held in the outer flame of an alcohol burner for 10 min before each measurement. In the surface tension test, equilibrium was considered achieved when the surface tension values stabilized within a constant range. Each concentration of C_8_P_x_E_y_C solutions was measured 3 times consecutively, and a standard deviation of less than 0.5 mN/m was ensured. The experiments were performed at 30 ± 0.5 °C.

#### 2.2.2. Contact Angle Measurement

The quartz plates were initially scrubbed thoroughly with a soft brush and laundry detergent. Next, they were rinsed sequentially with tap water, deionized water, and acetone. Then, the above quartz plates were rinsed with deionized water and dried. The dried quartz plates were treated in a chromic acid bath for 5 h to eliminate any potential organic contaminants. Subsequently, they were thoroughly rinsed with water and subjected to ultrasonication in ultrapure water for 20 min. Finally, the quartz was dried in an oven at 105 °C for contact angle tests.

We measured the contact angles on the quartz surface employing a LAUDA Scientific GmbH goniometer (LAUDA Scientific GmbH, Hirschhorn, Germany) and the sessile drop technique. A droplet of surfactant solution (2 μL) at a specified concentration was first deposited onto the cleaned solid surface using a syringe needle. The droplet profile was then captured by a camera, and the contact angles at different time points were determined through software analysis. In this work, the contact angle at 60 s after droplet deposition was specifically recorded. Measurements were repeated at multiple locations on the same quartz surface, and the reported contact angles are the average value of five results with a standard deviation of ≤2°.

## 3. Results

### 3.1. Surface Tension of C_8_P_x_E_y_C

**Figure 1:**
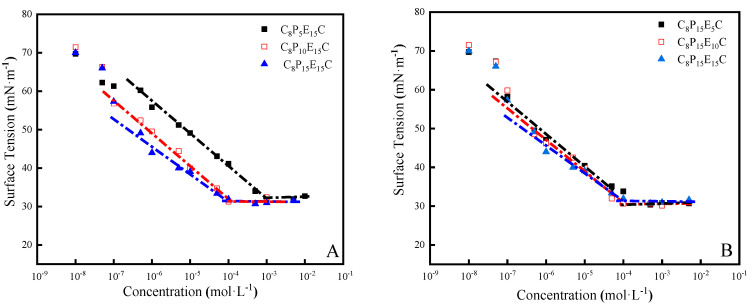
Relationship between surface tension ($\gamma_{LV}$) and the concentration of C_8_P_x_E_15_C (**A**) and C_8_P_15_E_y_C (**B**) aqueous solutions.

The adsorption of C_8_P_x_E_y_C at the air–water interface involves the process of nonpolar groups gradually displacing water molecules at the surface. This process governs most behaviors of extended surfactants when they contact solid surfaces. The $\gamma_{LV}$ values of five C_8_P_x_E_y_C surfactants with ultra-short hydrophobic chains are shown in Figure 1. By fitting the $\gamma_{LV}$ curves, the CMC value can be obtained at the turning point. It can be seen that $\gamma_{LV}$ gradually decreases with increasing concentration in Figure 1. Before CMC, the $\gamma_{LV}$-concentration curves exhibit a linear decreasing trend. After CMC, $\gamma_{LV}$ remains almost constant. Moreover, when PO units increase, the slope of $\gamma_{LV}$-concentration curves and the CMC value decrease in Figure 1A. This phenomenon is primarily due to the intrinsic hydrophobicity of PO units. The longer the PO chain, the higher the hydrophobicity of C_8_P_x_E_y_C molecules. Higher hydrophobicity facilitates molecular aggregation via hydrophobic interactions and makes it easier for C_8_P_x_E_y_C molecules to form micelles in solution. In contrast, the variation of EO groups has little effect on the slope in Figure 1B. This phenomenon may be attributed to the unique characteristic of the EO group. When the EO chain is short, the EO segments are extended, and the ether oxygen atoms are fully hydrated. Thus, hydrophilicity is directly determined by the EO chain length. When the EO chain is long, intramolecular hydrogen bonding leads the EO chain to coil or aggregate, reducing the effective hydration sites for hydrophilicity. As a result, the actual enhancement in hydrophilicity is much less than expected from the increase in EO chain length.

Two key parameters for quantifying surfactant adsorption at interfaces are the maximum surface excess concentration ($\Gamma_{\max}$) and the minimum molecular area ($A_{\min}$). These metrics are derived from the Gibbs adsorption isotherm, as shown in the following equations [25,26]:

(1) $$\Gamma_{\max}=\;-\;(\frac{1}{2.303nRT})(\frac{d\gamma }{\mathrm{dlog}C})$$

(2) $$A_{\min}=\frac{1}{N_{A}\Gamma_{\max}}\times 10^{14}$$

In these equations, γ denotes surface tension, *C* is the bulk concentration, *R* represents the universal gas constant (8.314 J·mol^−1^·K^−1^), *T* is the absolute temperature, N_A_ is Avogadro’s constant, and *n* in the equation is taken as 1. The adsorption parameters, including CMC, $\gamma_{CMC}$, *Г*_max_, and A_min_ for the five C_8_P_x_E_y_C molecules calculated using the Gibbs adsorption equation are listed in Table 1.

In Table 1, as the PO/EO chain length increases, the CMC value gradually decreases, the maximum saturated adsorption amount (*Γ*_max_) also decreases, and A_min_ gradually increases. This behavior is due to the enhanced hydrophobic interaction as the PO/EO chain length of the C_8_P_x_E_15_C molecules increases. As a result, the surfactants form micelles more readily in solution, leading to a lower CMC value. The saturated adsorption amount is determined by both the monomer concentration in the bulk solution and the interfacial adsorption equilibrium constant. An increase in the PO/EO chain length reduces the CMC, which in turn diminishes the available monomer for adsorption. Consequently, the surfactant monomers available for interface adsorption in solution decrease. Meanwhile, the enhanced hydrophobic interaction favors micelle formation. Under the combined effect of these two factors, the *Γ*_max_ of C_8_P_x_E_y_C at the interface decreases. Furthermore, a longer PO/EO chain length increases the interfacial size occupied by the hydrophilic chain. Thus, the surface area occupied per molecule increases, leading to a corresponding increase in the A_min_ value.

### 3.2. Contact Angles of C_8_P_x_E_y_C

**Figure 2:**
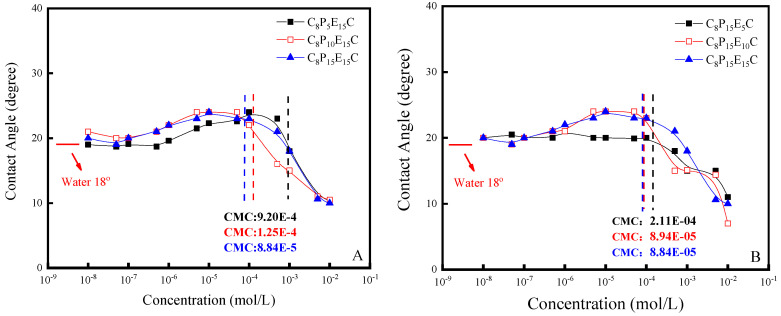
Contact angles of C_8_P_x_E_15_C (**A**) and C_8_P_15_E_y_C (**B**) on a quartz surface.

The variation in contact angles of C_8_P_x_E_y_C on a quartz surface as a function of concentration is depicted in Figure 2. The contact angle increases gradually before CMC under the combined effect of surface tension and interfacial tension, indicating an enhanced hydrophobicity of the quartz surface. After CMC, the surface tension remains constant. However, due to the changes in interfacial tension, the contact angle decreases sharply. Eventually, the contact angle stabilizes at a lower value, which reflects an enhancement in the hydrophilicity of the quartz surface. This agrees with the wetting behavior reported by Zhang et al. for cationic surfactants (C_16_PC, C_16_GPC, C_16_(EO)_3_PC, and C_16_G(EO)_3_PC) on quartz [10]. In addition, the contact angles of C_8_P_x_E_y_C increase with increasing PO units before CMC in Figure 2A.

### 3.3. Adhesional Tension of C_8_P_x_E_y_C

The adhesional tension ($\gamma_{\mathrm{LV}}\cos\theta$), which quantifies the work of adhesion, is defined as the difference between the surface free energy of quartz ($\gamma_{SV}$) and the quartz–liquid interfacial tension ($\gamma_{SL}$). The adhesional tension can be used to evaluate the adhesion capability of C_8_P_x_E_y_C at the quartz–liquid interface. According to Young’s equation and previous studies [30,31], certain surfactant solutions exhibit a linear correlation between $\gamma_{LV}$ and $\gamma_{\mathrm{LV}}\cos\theta$ on smooth and homogeneous solid surfaces.

(3) $$\gamma_{SV}-\gamma_{SL}=\gamma_{\mathrm{LV}}\cos\theta$$

(4) $${a\gamma }_{LV}+b=\gamma_{\mathrm{LV}}\cos\theta$$

In these equations, *a* and *b* are constant parameters. The value of a is intrinsically linked to the nature of the solid substrate. A combination of Young’s equation and the Gibbs adsorption isotherm allows for the quantification of the adsorption amount at both the quartz–liquid and air–liquid interfaces, as derived below [32]:

(5) $$\frac{d\left(\gamma_{LV}cos\theta \right)}{d\gamma_{LV}}=\frac{\Gamma_{SV}-\Gamma_{SL}}{\Gamma_{LV}}$$

(6) $$\frac{\Gamma_{SL}}{\Gamma_{LV}}=\left|a\right|$$

where *Г*_SV_, *Г*_SL_, and *Г*_LV_ are the saturated adsorption amounts at the solid–air, solid–liquid, and liquid–air interfaces, respectively. As extended surfactant molecules have no adsorption behavior at the quartz–air interface, *Г*_SV_ is 0. By fitting the linear part before CMC, the value of *Г*_SL_*/Г*_LV_ for C_8_P_x_E_y_C can be determined.

**Figure 3:**
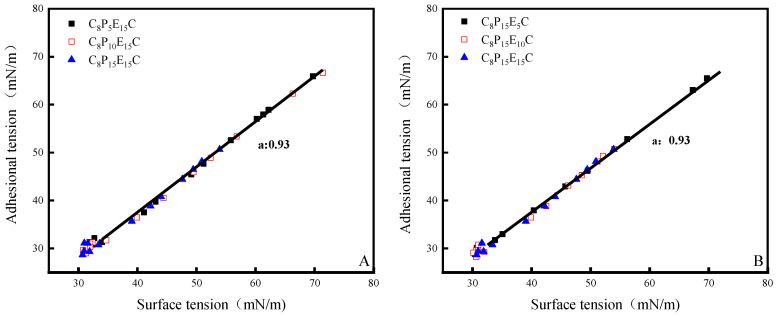
Relationship between adhesional tension ($\gamma_{\mathrm{LV}}\cos\theta$) and surface tension ($\gamma_{LV}$) for C_8_P_x_E_15_C (**A**) and C_8_P_15_E_y_C (**B**) solutions.

The variation of $\gamma_{\mathrm{LV}}\cos\theta$ as a function of $\gamma_{LV}$ for C_8_P_x_E_y_C solutions is depicted in Figure 3. Before CMC, $\gamma_{\mathrm{LV}}\cos\theta$ of the extended anionic surfactants with short hydrophobic chains exhibits a linear dependence on surface tension, which is in agreement with the results from Zdziennicka’s group [11,12]. Before CMC, both $\gamma_{LV}$ and $\gamma_{\mathrm{LV}}\cos\theta$ gradually decrease with increasing concentration. After CMC, the adsorption of C_8_P_x_E_y_C at the air–water interface reaches saturation. Thus, $\gamma_{LV}$ remains nearly constant, while $\gamma_{\mathrm{LV}}\cos\theta$ increases slightly with increasing concentration. It is known that the quartz surface is a high-energy surface. The extended anionic surfactants with short hydrophobic chains adsorb on the quartz surface by their EO groups via hydrogen bonding. As the hydrophobic part (C_8_P_n_) orients toward water, the quartz surface’s hydrophilicity reduces. It is noteworthy that this adsorption behavior differs from that of cationic surfactants. Cationic surfactants adsorb on quartz surfaces by electrostatic interactions [10].

As the concentration increases, C_8_P_x_E_y_C molecules adsorb on the quartz surface. At the CMC, a monomolecular adsorption layer is established by C_8_P_x_E_y_C surfactants on quartz. After CMC, there are still a small number of extended surfactant molecules that adsorb onto the pre-adsorbed monolayer via hydrophobic interactions. As their hydrophilic ionic heads point towards water, an adsorption bilayer with a loose structure is formed. Thus, $\gamma_{\mathrm{LV}}\cos\theta$ increases. This result is consistent with the trend of cationic surfactants reported by Zhang et al. [10]

By fitting the slope of the adhesional tension curve, the value of *Γ*_SL_/*Γ*_LV_ before CMC can be computed. As shown in Table 2, all C_8_P_x_E_y_C molecules exhibit positive *Γ*_SL_/*Γ*_LV_ values. As the PO/EO chain length increases, the adsorption amount decreases while the adsorption area increases. The CMC value decreases with increasing PO/EO chain length. Thus, the concentration of surfactant monomers available for adsorption at the quartz interface decreases. Simultaneously, the enhanced hydrophobic interactions promote the formation of micelles by these extended surfactants. Under the combined influence of these two factors, the saturated adsorption amount on the quartz surface decreases. Furthermore, longer PO/EO chain length increases the interfacial size occupied by the hydrophilic chain. As the surface area required per molecule increases, the A_min_ value increases correspondingly.

### 3.4. Interfacial Tension of C_8_P_x_E_y_C on the Quartz–Liquid Interface

**Figure 4:**
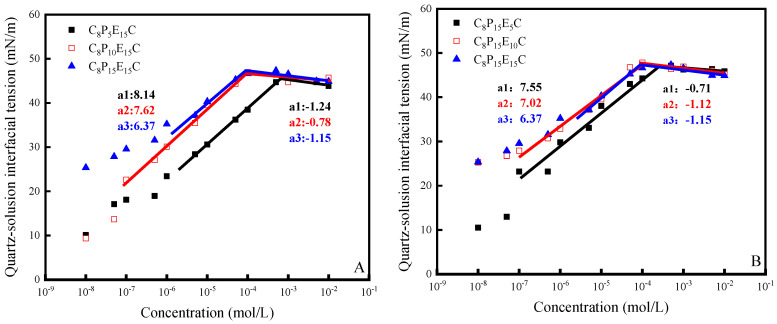
Relationship between quartz–liquid interfacial tension (γ_SL_) and concentration for C_8_P_x_E_15_C (**A**) and C_8_P_15_E_y_C (**B**).

The quartz–liquid interfacial tension is a critical parameter characterizing the adsorption behavior of C_8_P_x_E_y_C on quartz surfaces. Studying the quartz–solution interfacial tension ($\gamma_{SL}$) allows for a thorough understanding of the changes in quartz surface wettability induced by extended surfactants with an ultra-short hydrophobic chain. According to previous literature [33], the surface free energy of quartz is 76.42 mN/m. Values of γ_SL_ with concentration were calculated according to Young’s equation and plotted in Figure 4. Additionally, the slopes of the two stages in Figure 4 can be obtained by fitting the quartz–solution interfacial tension curves.

Based on results in Figure 4, the interfacial behaviors of these five extended surfactants with ultra-short hydrophobic chains (C_8_P_x_E_y_C) at the quartz–liquid interface are analyzed. The interfacial tension (γ_SL_) of C_8_P_x_E_y_C exhibits a typical concentration-dependent behavior in the two stages. Before CMC, γ_SL_ increases with increasing concentration, suggesting the adsorption of C_8_P_x_E_y_C and the reduction of quartz hydrophilicity. C_8_P_x_E_y_C surfactants adsorb on quartz via hydrogen bonding with hydrophobic group towards water, thereby decreasing the hydrophilicity of the quartz surface. After CMC, a distinct transition appears in the γ_SL_ concentration curve, implying that the adsorption mechanism of these extended surfactants changes. In this stage, C_8_P_x_E_y_C molecules in the bulk phase adsorb onto quartz by hydrophobic interactions with their hydrophilic heads oriented toward water. Therefore, the hydrophilicity of the quartz surface is enhanced. Notably, the PO chain plays a regulatory role in determining γ_SL._ Before CMC, longer PO chains lead to higher γ_SL_ values. The hydrophobicity of C_8_P_x_E_y_C molecules increases with PO chain length, and this promotes the anchoring of C_8_P_x_E_y_C surfactants on quartz. After CMC, the C_8_P_x_E_y_C molecule’s adsorption amount is small, and variations in PO chain length exert little influence on γ_SL_. Shorter EO chains reduce the hydrophilicity of C_8_P_x_E_y_C molecules, which promotes the insertion of hydrophobic alkyl groups into the interface. As the driving force for monolayer adsorption enhances, the interfacial tension exhibits a more pronounced increase with concentration. Therefore, the adsorption kinetics and interfacial activity of extended surfactants can be optimized by tuning the PO/EO chain lengths. This finding provides guidance for designing highly efficient extended surfactants with ultra-short hydrophobic chains.

In Table 3, the five C_8_P_x_E_y_C surfactants exhibit a distinct structure-dependent trend in their adsorption behavior on quartz. As the PO/EO chain length increases, the maximum saturated adsorption amount at the interface decreases. When the PO chain length increases, the hydrophobicity of C_8_P_x_E_y_C molecules enhances, and the molecular size of C_8_P_x_E_y_C increases. Thus, the interfacial film formed by C_8_P_x_E_y_C molecules becomes looser and each C_8_P_x_E_y_C molecule occupies a larger surface area, thereby reducing the saturated adsorption amount. When the EO chain length increases, the hydrophilicity of C_8_P_x_E_y_C molecules improves, which enhances the polar interaction between quartz and C_8_P_x_E_y_C molecules. Thus, the interfacial adsorption amount of C_8_P_x_E_y_C molecules decreases. Meanwhile, the C_8_P_x_E_y_C molecules’ adsorption area increases. By adjusting PO/EO chain lengths, the adsorption amount and molecular arrangement of C_8_P_x_E_y_C can be controlled to modify the interfacial property of the quartz surface. This regularity provides a basis for using functional surfactants to hydrophobically modify and control the wettability of quartz surfaces.

### 3.5. Adhesion Work of C_8_P_x_E_y_C

The adhesion work (*W_A_*), defined as the energy needed to separate a unit area of liquid from a solid, can be formulated via Young’s equation [34,35] as shown in Equation (8).

(7) $$W_{A}=\gamma_{SV}+\gamma_{LV}-\gamma_{SL}$$

(8) $$W_{A}=\gamma_{LV}(cos\theta +1)$$

**Figure 5:**
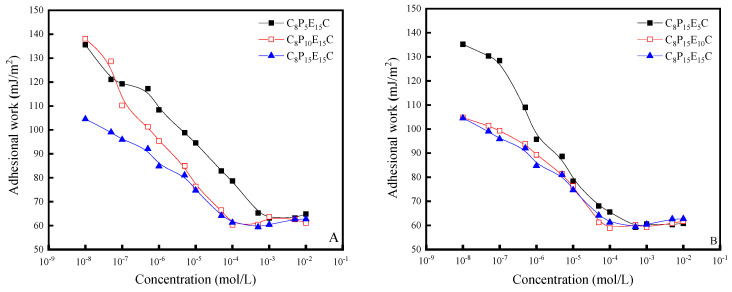
Relationship between adhesion work and concentration for C_8_P_x_E_15_C (**A**) and C_8_P_15_E_y_C (**B**).

The relationship between adhesion work and concentration for C_8_P_x_E_y_C is illustrated in Figure 5. Before CMC, as the adsorption of C_8_P_x_E_y_C at the liquid–air interface increases, $\gamma_{LV}$ and $\gamma_{\mathrm{LV}}\cos\theta$ decrease with concentration. As a result, $W_{A}$, which is governed by both surface tension and adhesion tension, decreases with increasing concentration. After CMC, C_8_P_x_E_y_C adsorbs at the air–liquid interface and reaches saturation when the bulk concentration increases further. At this stage, $\gamma_{LV}$ remains constant. As a turning point appears in $W_{A}$, $\gamma_{\mathrm{LV}}\cos\theta$ controls the subsequent variation of adhesion work predominantly. For the five C_8_P_x_E_y_C solutions, $W_{A}$ reaches a minimum value near their CMC values. This minimum $W_{A}$ corresponds to the onset of a loose bilayer structure formed by C_8_P_x_E_y_C on quartz. $W_{A}$ gradually decreases as PO units increase at high concentration, whereas the variation in EO chain length has little influence on $W_{A}$.

### 3.6. Adsorption Mechanism of C_8_P_x_E_y_C on Quartz

**Figure 6:**
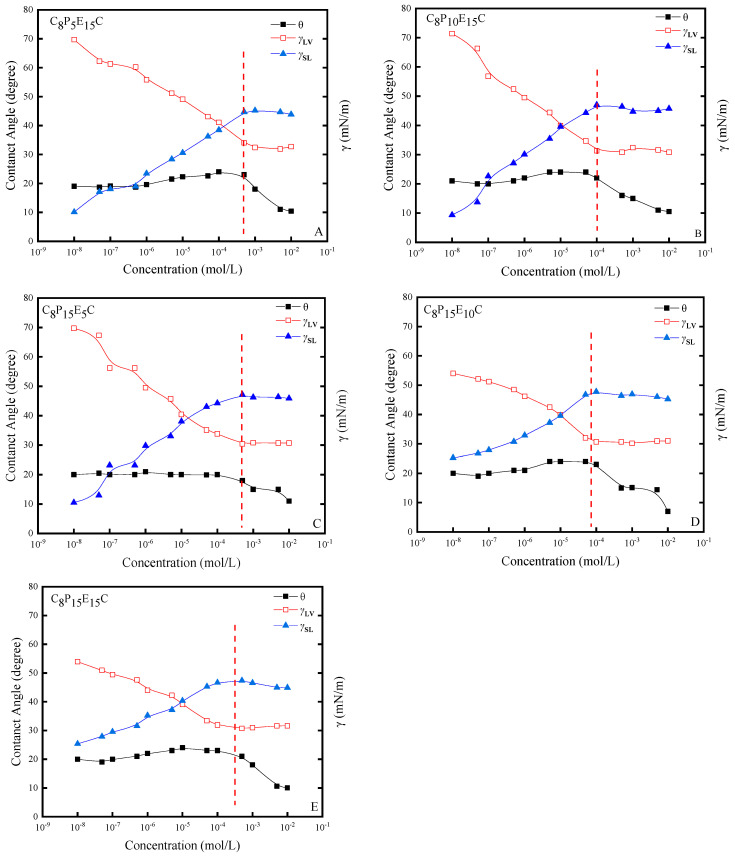
Adsorption mechanism of C_8_P_5_E_15_C (**A**), C_8_P_10_E_15_C (**B**), C_8_P_15_E_5_C (**C**), C_8_P_15_E_10_C (**D**), and C_8_P_15_E_15_C (**E**) on quartz.

**Figure 7:**
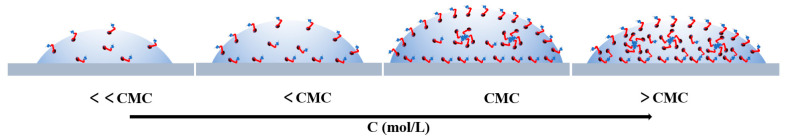
Possible adsorption behaviors of C_8_P_x_E_y_C on the quartz surface.

The dependencies of surface tension, contact angle, and interfacial tension on concentration are plotted in Figure 6. Clearly, the changes in contact angle, surface tension, and interfacial tension can be divided into two stages. A possible schematic illustration of the adsorption behaviors for C_8_P_x_E_y_C on the quartz surface is provided in Figure 7.

Interfacial adsorption refers to the enrichment of surfactant molecules at the air–liquid and solid–liquid interfaces. By reducing interfacial energy, wetting, emulsification, and dispersion can be achieved by surfactant molecules. The adsorption behavior is closely related to C_8_P_x_E_y_C solutions’ concentration, and the CMC value of C_8_P_x_E_y_C is a critical transition point for adsorption. Before CMC, surfactants form a monolayer on quartz. After CMC, micelles are formed in the solution, and surfactant molecules adsorb on quartz to form a loose bilayer structure. As shown in Figure 6, the adsorption process of C_8_P_x_E_y_C has two stages.

In the first stage, C_8_P_x_E_y_C adsorbs at the air–liquid interface via hydrophobic interactions at low concentrations, which leads to a decrease in surface tension. Simultaneously, C_8_P_x_E_y_C adsorb on quartz by hydrogen bonding with their hydrophobic tails oriented toward water, which results in a slow increase in interfacial tension. The contact angle increases slightly due to the combined effects of surface tension and interfacial tension, indicating the reduced hydrophilicity of quartz. As the C_8_P_x_E_y_C solution’s concentration increases, C_8_P_x_E_y_C continues to adsorb at both the air–liquid and quartz–liquid interfaces. At CMC, C_8_P_x_E_y_C molecules adsorb to saturation at both the air–liquid and quartz–liquid interfaces.

In the second stage, the adsorbed C_8_P_x_E_y_C at the air–liquid interface reaches saturation. As the concentration of the C_8_P_x_E_y_C solution increases further, micelles are formed in the solution. Accordingly, the surface tension of C_8_P_x_E_y_C solutions remains nearly constant. At the quartz–liquid interface, a small number of C_8_P_x_E_y_C molecules adsorb onto the pre-adsorbed monolayer formed by C_8_P_x_E_y_C molecules through hydrophobic interactions. Thus, a loose bilayer structure is formed with the hydrophilic heads of C_8_P_x_E_y_C molecules towards water. Consequently, the interfacial tension decreases, the contact angle reduces, and the quartz surface’s hydrophilicity improves.

## 4. Conclusions

Herein, the adsorption behaviors of five short hydrophobic extended anionic surfactants (C_8_P_x_E_y_C) on the quartz surface were investigated. The main findings are

(1) The adsorption process exhibits two distinct stages. Before CMC, C_8_P_x_E_y_C molecules form a monolayer at the quartz–liquid interface via hydrogen bonding with their hydrophobic tails oriented toward the aqueous phase, thereby reducing the hydrophilicity of the quartz surface. After CMC, a loose bilayer structure forms through hydrophobic interactions, with hydrophilic heads facing the aqueous phase, which enhances the hydrophilicity of the quartz surface.

(2) Longer PO chains enhance hydrophobicity and size of C_8_P_x_E_y_C molecules, leading to a looser interfacial packing and a decrease in the saturated adsorption amount. Longer EO chains improve hydrophilicity of C_8_P_x_E_y_C molecules, strengthening polar interactions with the quartz surface and resulting in a decreased interfacial adsorption amount and an increased molecular area.

These findings provide insights for designing efficient short hydrophobic extended anionic surfactants. The ability to precisely control quartz wettability through molecular structure makes these extended surfactants promising for practical applications in enhanced oil recovery, material coatings, and other industrial processes where interfacial control is crucial.

## Figures and Tables

**Table 1:** The CMC, γ_CMC_, *Г*_max_, and A_min_ values of C_8_P_x_E_y_C.

Molecular Formulas	CMC (mol·L^−1^)	γ_CMC_ (mN·m^−1^)	*Г*_max_ (10^−10^ mol·m^−2^)	A_min_ (nm^2^)
C_8_P_5_E_15_C	9.20 × 10^−4^	32.18	1.50	1.11
C_8_P_10_E_15_C	1.25 × 10^−4^	31.23	1.47	1.12
C_8_P_15_E_5_C	1.43 × 10^−4^	30.56	1.43	1.16
C_8_P_15_E_10_C	9.71 × 10^−5^	30.84	1.39	1.19
C_8_P_15_E_15_C	8.84 × 10^−5^	31.37	1.26	1.32
C_16_E_3_C [27]	9.40 × 10^−5^	22.82	1.73	0.96
C_16_E_5_C [27]	4.50 × 10^−5^	31.84	1.52	1.09
C_16_E_7_C [27]	1.00 × 10^−5^	34.69	1.31	1.31
C_12_P_4_S [28]	4.20 × 10^−4^	40.00	1.66	1.66
C_12_P_8_S [28]	7.80 × 10^−5^	35.50	1.54	1.54
C_12_P_12_S [28]	4.30 × 10^−5^	33.70	1.64	1.64
S-C_12_P_7_S [29]	4.60 × 10^−5^	33.80	1.47	1.47
S-C_12_P_13_S [29]	1.20 × 10^−5^	31.90	1.48	1.48

**Table 2:** The value of *Γ*_SL_/*Γ*_LV_ for C_8_P_x_E_y_C before CMC.

Surfactants	*Г*_SL_/*Г*_LV_	*Г*_max_ (10^−10^ mol·m^−2^)	A_min_ (nm^2^)
C_8_P_5_E_15_C	0.93	1.40	1.19
C_8_P_10_E_15_C	0.93	1.37	1.21
C_8_P_15_E_5_C	0.93	1.33	1.25
C_8_P_15_E_10_C	0.93	1.29	1.29
C_8_P_15_E_15_C	0.93	1.17	1.42

**Table 3:** Adsorption parameters of C_8_P_x_E_y_C on quartz.

Abrr.	*Γ*_below_ (10^−10^ mol·m^−2^)	A_below_ (nm^2^)	*Γ*_above_ (10^−10^ mol·m^−2^)	A_above_ (nm^2^)
C_8_P_5_E_15_C	1.43	1.16	0.22	7.64
C_8_P_10_E_15_C	1.34	1.24	0.14	12.15
C_8_P_15_E_5_C	1.32	1.26	0.12	13.35
C_8_P_15_E_10_C	1.23	1.35	0.20	8.46
C_8_P_15_E_15_C	1.12	1.49	0.20	8.24

## Data Availability

The original contributions presented in this study are included in this article. Further inquiries can be directed to the corresponding author.
